# Pleomorphic Adenoma of the Palate With an Atypical Malignant Presentation: A Case Report

**DOI:** 10.7759/cureus.42365

**Published:** 2023-07-24

**Authors:** Mandeep Singh, Ejaz A Mokhtar, Shahrukh Akbar, Peeyush Shivhare, Ameera Salahudheen

**Affiliations:** 1 Oral and Maxillofacial Surgery, Dr. B.R. Ambedkar Institute of Dental Sciences and Hospital, Patna, IND; 2 Oral and Maxillofacial Surgery, All India Institute of Medical Sciences, Patna, IND; 3 Dentistry, College of Dental Sciences, Patna, IND; 4 Dentistry, All India Institute of Medical Sciences, Patna, IND

**Keywords:** pleomorphic adenoma, minor salivary gland, excision, ulceration, malignancy, palate

## Abstract

A pleomorphic adenoma is a mixed salivary gland tumor. The parotid gland is the most common site. The intraoral palate is the most common site due to the abundance of accessory salivary glands in the palatal area. It has a very slow growth rate and is usually painless. Consequently, patients often have a lengthy history of presentation.

Herein, we report a case of pleomorphic adenoma of the palate in a 53-year-old male patient. The mass was 5 cm by 4 cm in size. The growth rate was rapid, and it attained a large size in just nine months. On clinical examination, the overlying mucosa was ulcerated. On general examination, lymphadenopathy of the right side level 1 b lymph node was found. These clinical findings were consistent with malignancy. However, the histopathological report negated the clinical findings of malignant salivary gland tumors. The tumor was managed with a wide local excision of the tumor with a 1 cm clear margin. The postoperative course was uneventful. No recurrence was seen after two years of follow-up. A thorough cytological or histological examination is a prerequisite to defining the malignant nature of the lesion.

## Introduction

Pleomorphic adenoma is the most common tumor of the salivary gland. It affects both major and minor salivary glands. It mostly affects the major salivary glands. The parotid is the most common site, with an incidence of 75%. Minor salivary glands account for around 7% of the affected glands. Intraorally, the palatal salivary glands are most commonly affected, with an incidence of about 45%. It has a female predilection, with an age distribution of 20 to 75 years [1, 2].

Clinically, it presents as a firm, painless submucosal mass. It has a very slow growth rate, increasing in size slowly over the years. The overlying mucosa is mostly intact, with a few cases reporting ulcerated mucosa [3]. The ulceration in the epithelium is usually seen due to trauma to the tumor or if the patient had earlier gone for a biopsy.

Minor salivary gland carcinoma accounts for more than half of all salivary gland carcinomas reported in the head and neck. Around 60%-80% of reported minor salivary gland tumors are malignant [4-6]. These data, along with the clinical findings, give rise to the suspicion that the tumor is malignant.

The aim of this paper is to report an unusual clinical presentation of pleomorphic adenoma of the palate with ulceration of the overlying mucosa and a rapid growth rate. The clinician should be aware of these rare clinical findings and wait for the histopathological examination before making a final diagnosis.

## Case presentation

A 53-year-old male patient reported a mass on the right side of the palate for nine months. The mass had been slowly growing for the first six months, but the growth was very rapid in the last three months. The mass became ulcerated and painful in the last month. There were no other symptoms like numbness, dysphagia, stridor, or speech difficulties.

Past medical history revealed the patient was hypertensive and was on medications. Personal history revealed that the patient was a tobacco chewer. There was no previous history of biopsy or trauma to the mass. On lymph node examination, lymphadenopathy was seen in the right submandibular region.

**Figure 1:**
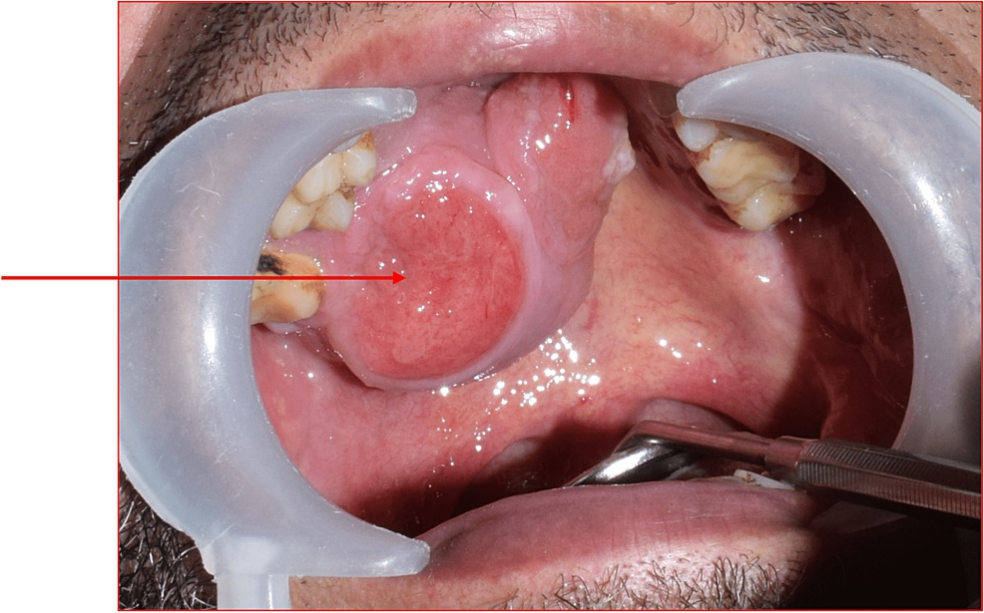
A clinical photograph showing an intraoral mass with ulcerated mucosa

A clinical intraoral examination detected a well-circumscribed mass covering the hard and soft palates. The mass was mostly present on the right side of the palate. It was 5 cm by 4 cm in diameter. It was somewhat spherical in shape with a well-defined edge. The mass was translucent. As the overlying mucosa was ulcerated (Figure 1), it was painful on palpation.

**Figure 2:**
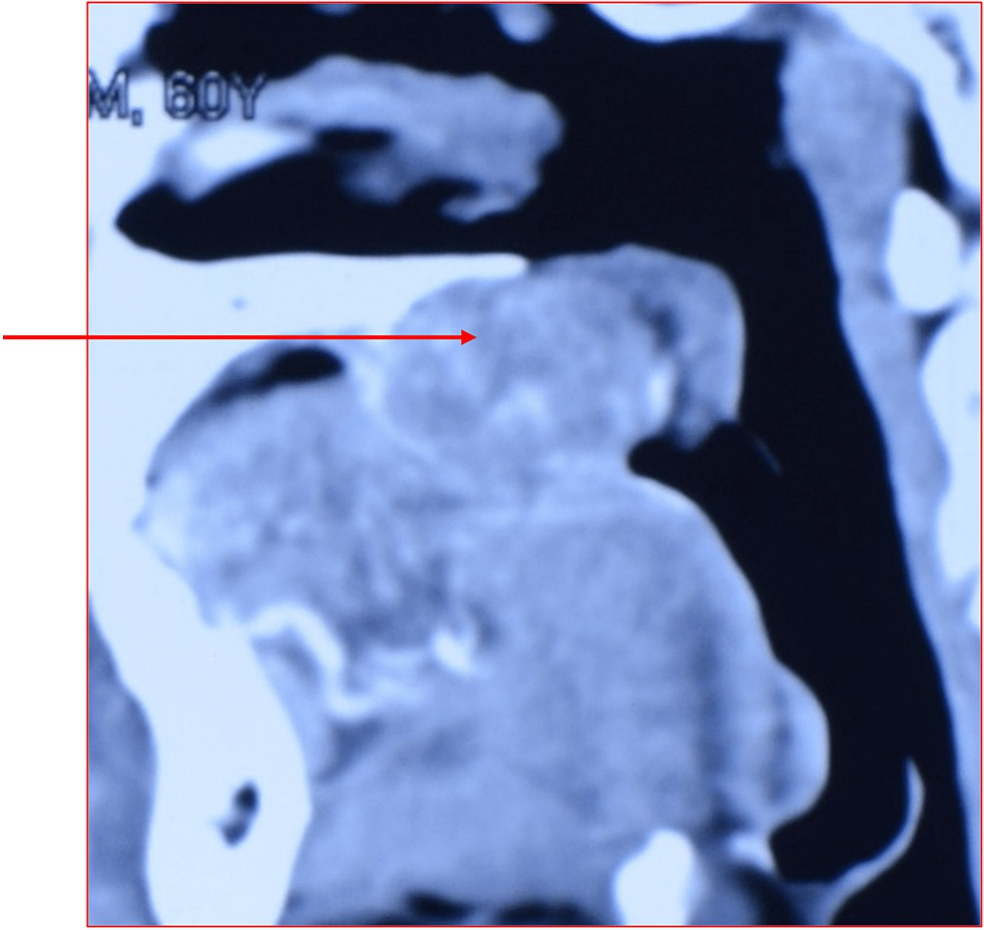
Sagittal-reconstructed CT image showing the mass causing cupping resorption of the hard palate

The temperature of the overlying mucosa was normal, and its consistency was firm. The mass was immobile, and no pulsation was felt. A computed tomography scan revealed a 4.5 cm x 4cm x 3 cm mass involving the right side of the palatal bone. The mass was well-circumscribed, and it was extending into posterior airway space. The mass caused cupping and resorption of the hard palate. However, a thin layer of palatal bone was intact (Figure 2).

The rapid growth of the tumor, along with ulceration without any history of trauma or biopsy, raised suspicion of malignancy. Fine needle aspiration cytology (FNAC) of the mass showed epithelial components in tubules and trabeculae with hyperkeratotic and hyperplastic squamous epithelium. However, no malignant cells were seen. The FNAC of the enlarged lymph node showed inflammatory changes.

**Figure 3:**
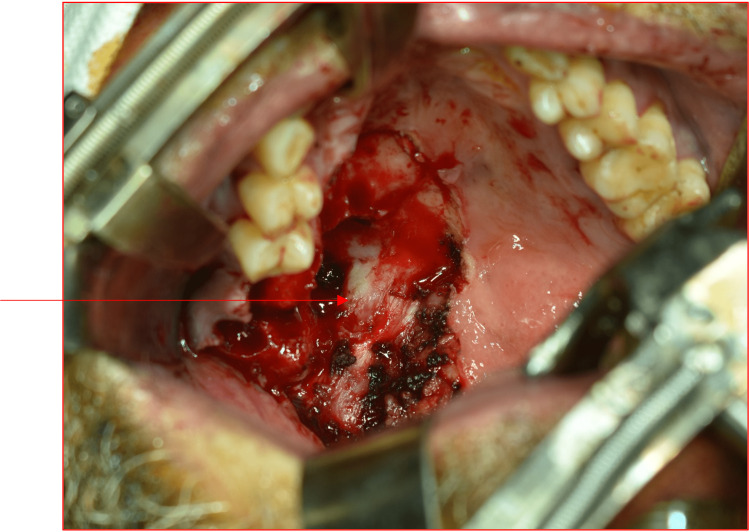
An intraoperative picture showing the excision of the tumor with overlying mucosa

After a routine preoperative blood examination and pre-anesthetic checkup, an excision of the tumor was planned. Under general anesthesia, the excision of the tumor with periosteum was carried out with overlying mucosa and a 1 cm clear margin (Figure 3).

**Figure 4:**
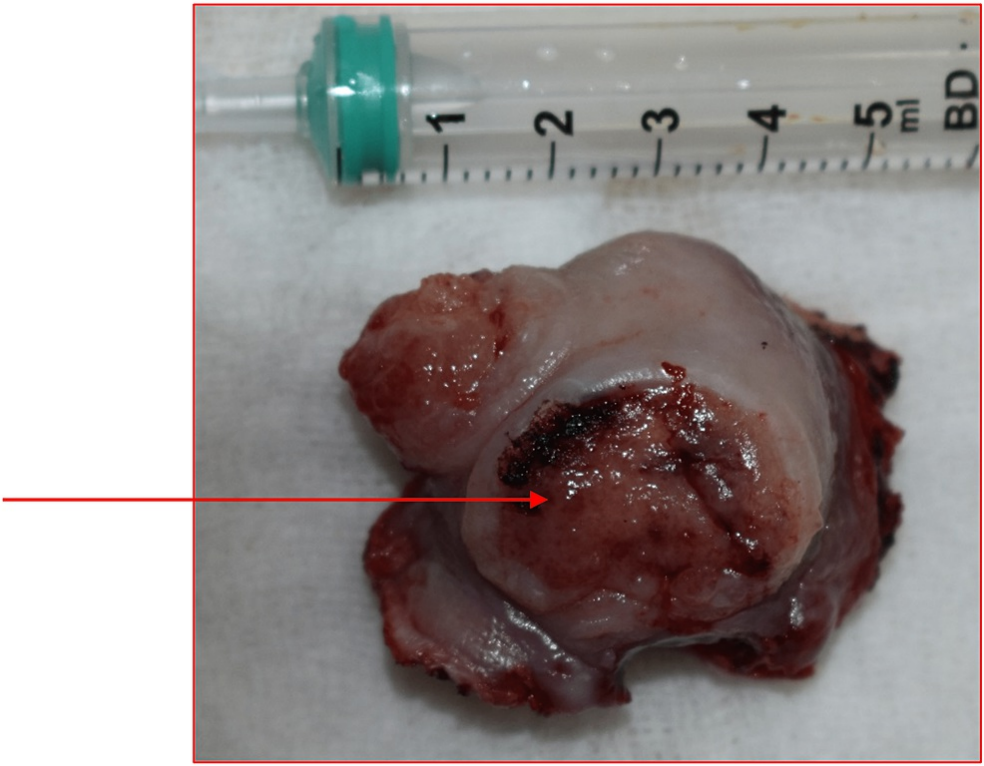
The arrow shows the excised mass having an ulcerated mucosa

The excised sample, as seen in Figure 4, was sent for histopathological examination.

**Figure 5:**
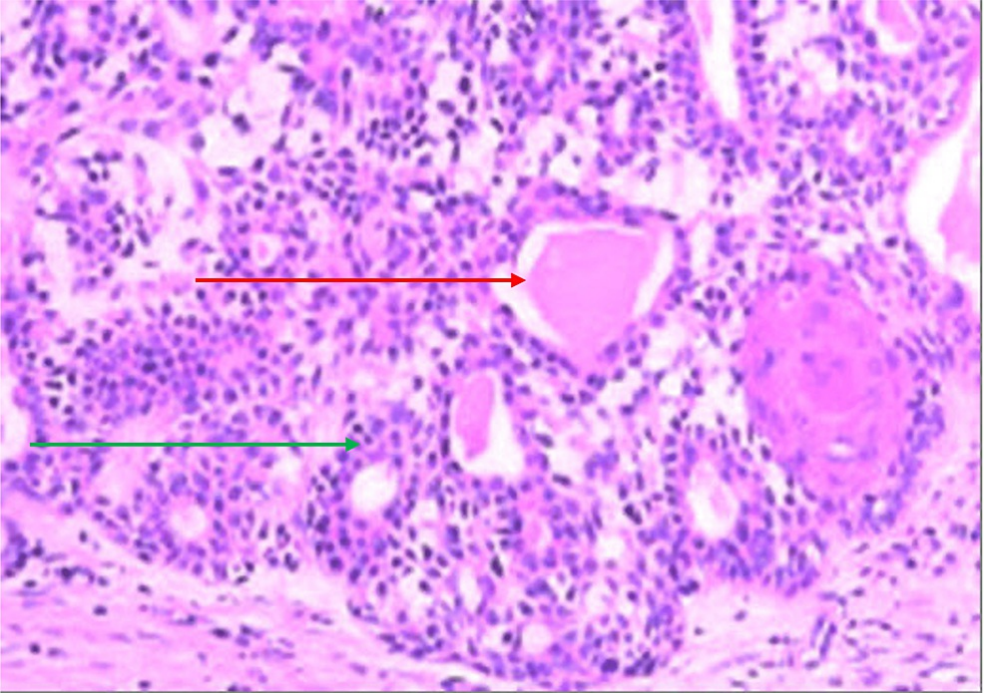
Histopathological examination of the sample. The green arrow shows myoepithelial cells, and the red arrow shows ductal cells.

Histopathological examination revealed that the tumor was biphasic in nature. It was composed of both epithelial and stromal components. Epithelial components were arranged in tubules, trabeculae, and cords. The cellular components were composed of epithelial and myoepithelial cells. Stroma was myxoid and showed adipocytes. The focal melting of epithelial components into the stroma was identified. This confirmed the diagnosis of pleomorphic adenoma. No malignant cells were seen (Figure 5).

**Figure 6:**
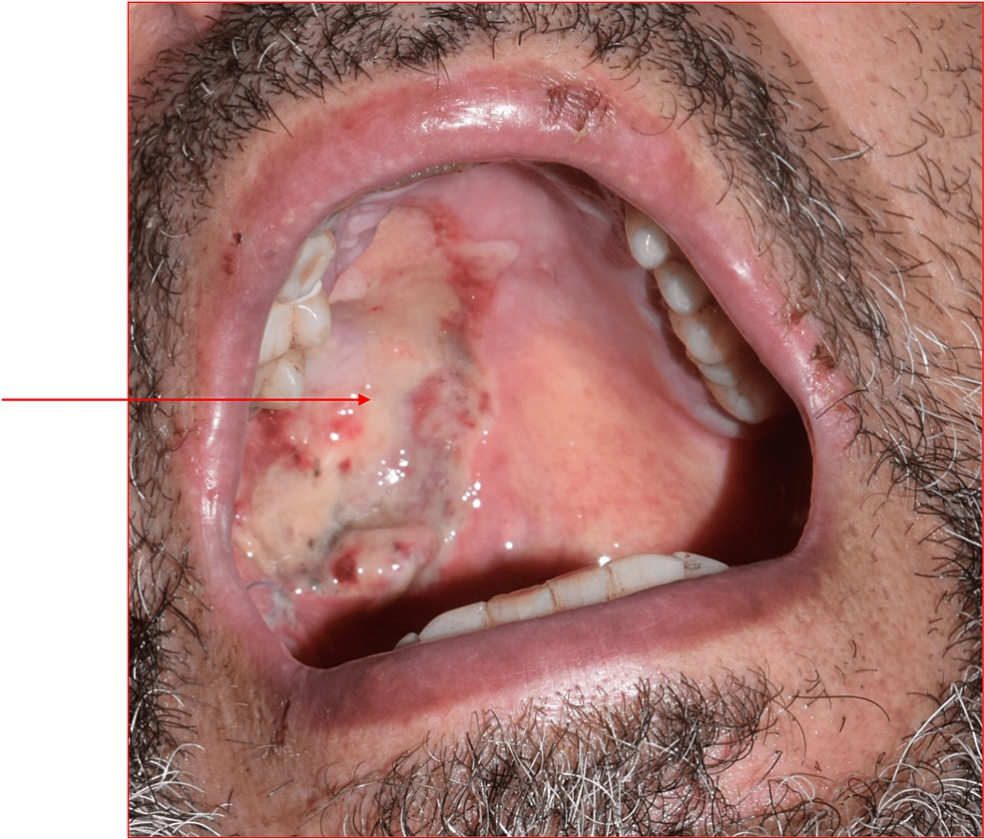
The patient's 15-day follow-up showed soft tissue covering the bared bone

Immunohistochemistry showed negative results for CD 117, CD 43, smooth muscle actin (SMA), and glial fibrillary acidic protein (GFAP), which ruled out malignant transformation. The patient's postoperative period was uneventful. After 15 days, soft tissue covered the bared bone (Figure 6).

**Figure 7:**
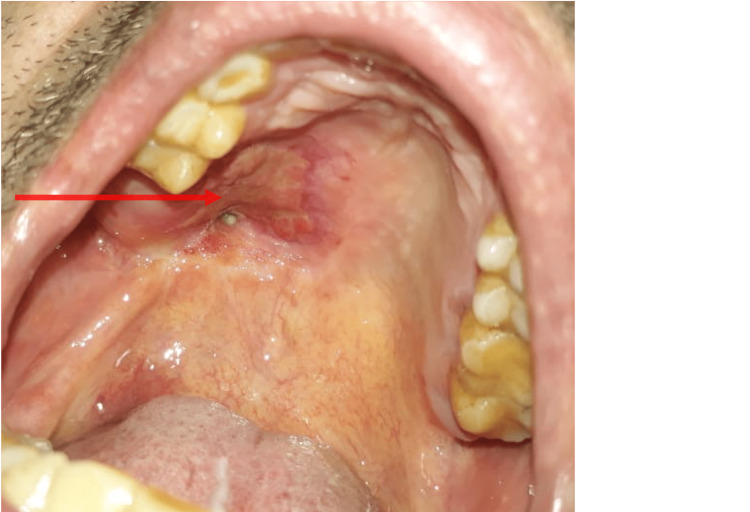
A postoperative six-month follow-up showed a significant amount of healing

A significant amount of healing was seen in six months (Figure 7).

The patient is under regular follow-up, and there is no evidence of recurrence after two years of follow-up.

## Discussion

The minor salivary glands are smaller salivary glands that are distributed across the upper aerodigestive tract. They numbered between 450 and 1000, and they are mostly present around the oral cavity [7]. Minor salivary gland carcinomas usually develop in the palatal (46.5%), buccal (9.4%), retromolar (3.6%), and labial salivary glands (2.4%) [8].

The malignant tumor is known to occur in the hard and soft palates. Pain and ulceration are usually the first clinical findings. The pain usually results from the local extension of the neoplasm into adjacent hard and soft tissue. The other findings are rapid growth and lymphadenopathy [9,10]. In our case, the patient had ulceration, pain, lymphadenopathy, and rapid growth. All these findings raised the suspicion of a malignant tumor.

Imaging plays a crucial role in the diagnosis of benign and malignant salivary gland tumors. Benign tumors mostly have smooth margins. Malignant salivary gland tumors usually have an ill-defined margin, and they show destructive growth and invasion into adjacent soft tissue or underlying bone. However, the imaging characteristics may vary depending on the histological grade of the salivary tumors. Low-grade salivary tumors also have smooth borders and may cause pressure erosion of adjacent bone, like benign tumors. However, high-grade tumors have irregular borders [11]. Therefore, merely by analyzing imaging, we cannot predict benign or malignant tumors.

Histologically, palatal tumors are mixed tumors composed of both ductal epithelium and myoepithelium. The tumor is surrounded by a fibrous tissue called a pseudocapsule [12]. The pseudocapsule is continuous in pleomorphic adenoma when it presents in the major salivary gland. However, when the tumor is present in the minor salivary gland, the pseudocapsule may be incomplete in some places [13,14].

The treatment of palatal pleomorphic adenoma is controversial. Some authors suggest the enucleation of the tumor. The advantage of enucleation of tumors is that it helps shave the overlying mucosa. However, the majority of authors suggest a wider local excision of the tumor. This is because of the uneven thickness of the pseudocapsule. The pseudocapsule is substantially thick in some places; it is thin and delicate in other sites, while in other places it is incomplete. This means that where the pseudocapsule is absent or incomplete, the normal tissue and tumor tissue are in close contact. Therefore, although the tumor tissue is clinically shelled out after enucleation, it may be left behind adhering to the normal tissue. Wu et al. observed on histopathological examination of 74 palatal pleomorphic adenomas that the tumor can be fully encapsulated (16%), partially encapsulated (54%), or unencapsulated (30%) [1]. Other features, like splits or cleavages within the tumor and pseudopods, also invite recurrence. Hence, simple enucleation should not be performed.

The above characteristics of the tumor strongly suggest wide local excision of the tumor with a clear margin. A clear margin of 1 cm is recommended by most surgeons. Excision or scraping of the palatal bone is not required, as pleomorphic adenoma does not release any osteoclast-activating factors to invade bone. Moreover, the periosteum is an effective anatomical barrier.

## Conclusions

The presence of ulceration and rapid growth in palatal pleomorphic adenoma usually suggests malignancy. However, these clinical findings do not guarantee malignancy. Histological examination is the gold standard for diagnosis. We should wait for a histopathological examination before making a final diagnosis based only on clinical findings. A wide local excision with a clear margin of 1 cm is an ideal surgical technique to prevent recurrence.
